# Effects of Medetomidine–Butorphanol and Medetomidine–Buprenorphine on Oxidative Stress and Vital Parameters in Dogs Undergoing Ovariohysterectomy

**DOI:** 10.3390/ani14091349

**Published:** 2024-04-30

**Authors:** Evelina Burbaitė, Sandra Čechovičienė, Ieva Sarapinienė, Birutė Karvelienė, Vita Riškevičienė, Gintaras Daunoras, Dalia Juodžentė

**Affiliations:** 1 Dr. L. Kriaučeliūnas Small Animal Clinic, Faculty of Veterinary, Veterinary Academy, Lithuanian University of Health Sciences, Tilžės str 18, 44307 Kaunas, Lithuania; 2 Neurology and Neurosurgery Division, San Marco Veterinary Clinic, 35030 Veggiano, Italy; 3 Institute of Cardiology, Lithuanian University of Health Sciences, Sukilėlių Ave 15, 44307 Kaunas, Lithuania; 4 Department of Veterinary Pathobiology, Faculty of Veterinary, Veterinary Academy, Lithuanian University of Health Sciences, Tilžės str 18, 44307 Kaunas, Lithuania; vita.riskeviciene@lsmu.lt

**Keywords:** anesthesia, buprenorphine, butorphanol, dog, oxidative stress

## Abstract

**Simple Summary:**

Oxidative stress is a metabolic state in which the organism possesses more oxidative compounds than antioxidant ones. This imbalance contributes to the formation of multiple pathologies and can be caused by different methods of drug administration, surgery, and anesthesia. The aim of the current study was to evaluate the effect of two different anesthetic protocols on oxidative stress and vital parameters. Ten dogs were randomly assigned into two groups and received different anesthetic agents during anesthesia for female spaying procedures. It was noted and concluded that the administration of medetomidine–butorphanol contributed to higher values of oxidative stress markers during different time frames when compared to the medetomidine–buprenorphine group. Even though the cardiorespiratory parameters were stable throughout the study in all dogs, the medetomidine–butorphanol group experienced greater alterations in vital parameters during anesthesia. Therefore, this study suggests that medetomidine–buprenorphine might be a superior anesthetic combination in comparison to medetomidine–butorphanol.

**Abstract:**

Oxidative stress (OS) is caused by an imbalance between the production of oxygen-containing free radicals and their elimination. General anesthesia increases the production of reactive oxygen species (ROS) and therefore causes oxidative stress. Our objective was to determine the effects of medetomidine–butorphanol (MEDBUT) and medetomidine–buprenorphine (MEDBUP) on oxidative stress and cardiorespiratory parameters in dogs undergoing ovariohysterectomy (OHE). Ten healthy female dogs were randomly assigned to two groups: the MEDBUT group (*n* = 5) received medetomidine and butorphanol, while the MEDBUP group (*n* = 5) received medetomidine and buprenorphine. OS was evaluated by measuring total antioxidant status (TAS), total oxidant status (TOS), and oxidative stress index (OSI) during five different time points (from the administration of anesthetic drugs to 2 h after surgery). The observed vital cardiorespiratory parameters included heart rate (HR), respiratory rate (fR), noninvasive systolic (SAP) and diastolic (DAP) arterial blood pressures, oxygen saturation (SpO_2_), end-tidal CO_2_ (EtCO_2_), and body temperature (BT). Cardiorespiratory parameters were altered at a significantly greater degree in animals sedated with MEDBUT (*p* < 0.05). The administration of medetomidine–butorphanol was more likely to increase OS parameters, while medetomidine–buprenorphine showed decreased levels of oxidative stress throughout the study.

## 1. Introduction

The loss of homeostasis between antioxidant and pro-oxidant properties inside the organism results in oxidative stress (OS). Reactive oxygen species (ROS) are essential for normal metabolism, as they are responsible for pathogen destruction and play a fundamental role in the inflammation process [1,2,3,4]. A loss of balance and the overproduction of reactive oxygen species can lead to oxidative stress that is linked to accelerated aging and the development of cardiovascular, dermatologic, and oncologic diseases in multiple species [1,2,3,4,5,6,7,8,9]. The overload of oxidants delays wound healing by displaying cytotoxic effects on dermal fibroblasts and keratinocytes, inactivating antioxidants, and stimulating inflammatory cell chemotaxis [4,9]. Recent studies have aimed to link oxidative stress with different drugs, surgical procedures, and anesthetic protocols, but the results are often controversial. A wide variety of diseases in human patients are proven to cause a lower antioxidant capacity [7,10,11]. In dogs, however, studies are still lacking. Different drugs were evaluated as oxidative stress causers. Anesthetics and opioids used for sedation and analgesia are some of them [12]. It is known that the severity of surgical intervention is positively correlated with OS [13]. Even the severity of pneumoperitoneum, which is necessary for laparoscopic procedures, elevates OS [14,15]. While it is not yet completely understood, evidence suggests that antioxidant supplementation might not prevent oxidative damage [16]. As mentioned above, research results regarding oxidative stress are often controversial. So, if other aspects are evaluated, overall, laparoscopic surgery, both in humans and dogs, can result in lesser oxidative stress, as the procedure is shorter, requires a shorter anesthesia time, and is less invasive [17,18]. Elevated levels of OS have also been associated with different anesthetic agents [19,20,21]. Some of them are thought to have antioxidant properties, while some of them act as pro-oxidants [22]. For example, propofol has been studied extensively because its molecular structure is similar to the powerful antioxidant tocopherol. Therefore, it has been speculated and proven that propofol has antioxidant properties as well [2,18,19,23,24,25]. Volatile anesthetics, like isoflurane or sevoflurane, have been proven to be antioxidants with anti-inflammatory properties, too [1,19,23,26,27]. Butorphanol has antioxidant properties in dogs as well as racoons [22,28,29]. Butorphanol and fentanyl combination was even shown to contribute to the protection of myocardial ischemia/reperfusion injury, counteracting oxidative stress in rats [30]. Buprenorphine, on the other hand, was proven to be both an antioxidant and causative of severe oxidative activity [6,20,22,31,32].

Currently, butorphanol and buprenorphine are considered two of the most popular and largely available pharmacological choices for multimodal sedation and moderate analgesia in veterinary medicine. Adding an opioid drug into the anesthetic protocol allows the dosage of medetomidine to be reduced and also potentiates its sedative and analgesic effects. The action of butorphanol and buprenorphine has been previously compared in studies. It was concluded that buprenorphine is the superior analgesic in dogs; therefore, it is used more commonly in surgical procedures [12,29]. Adverse effects observed after opioid administration are respiratory depression and mild cardiovascular changes such as bradycardia and hypotension [12].

Overviewing the scientific literature, it is noted that oxidative stress can be caused or inhibited by multiple factors; therefore, particular care and attention must be taken to ensure equal conditions in study objects. The evaluation of anesthesia depth and cardiorespiratory parameters is one of the ways to ensure the safety of anesthetic procedures [1,33]. Plasma total oxidant status (TOS), total antioxidant status (TAS), and oxidative stress index (OSI) were chosen to evaluate the oxidative stress status of the dogs undergoing OHE. TOS has been known as a parameter indicating cumulative oxidants in plasma, and TAS is a measurement that represents antioxidant properties and measures the ability to scavenge free radicals [2,34]. The OSI is also represented as a ratio between TOS and TAS that indicates the balance between oxidants and antioxidative properties [2,11,13,15,17,34].

Controversial results regarding the antioxidant and anesthetic properties of butorphanol and buprenorphine have led to our further investigation. The aim of this study was to determine the influence of two different anesthetic protocols (MEDBUT or MEDBUP) on oxidative stress and vital parameters in healthy anesthetized dogs during ovariohysterectomy (OHE).

## 2. Materials and Methods

### 2.1. Animals and Drugs

A total of 10 client-owned female dogs, scheduled for routine OHE surgery, were used in this study. The dogs were 18.10 ± 8.10 months old and weighed 23.00 ± 5.10 kg. All dogs belonged to medium-sized breed classes: 5 mixed-breed dogs, 2 Belgian Shepherds, 1 Mittelschnauzer, 1 East Siberian Laika, and 1 German Shorthaired Pointer. For inclusion, all dogs had to be classified as class I by the American Society of Anesthesiologists (ASA), based on a physical examination, complete blood cell count, the analysis of serum biochemistry, and thoracic radiographs.

The animal study protocol was approved by the Ethics Committee of the Lithuanian University of Health Sciences (protocol code Nr. 2024-BEC3-T-001, approved on 2nd of April 2024). All owners signed an informed consent form before enrolling the dogs in the study.

Dogs were randomly assigned into two different groups: the MEDBUT group (*n* = 5) received butorphanol (BUT) (0.14 mg kg^−1^) and the MEDBUP group (*n* = 5) received buprenorphine (BUP) (0.02 mg kg^−1^) (Richter Pharma AG, Wels, Austria). Both groups received 0.014 mg kg^−1^ medetomidine (MED) (CP Pharma Handelsgesellschaft mbH, Burgdorf, Germany). Dogs were fasted for 12 h before anesthesia. Drugs were administered intramuscularly in both groups. After the injection of sedatives, the dogs were then kept in the same quiet room for 15 min under the direct supervision of an anesthesiologist. An intravenous catheter was then placed in the left cephalic vein, and anesthesia was induced with propofol (PRO; Fresenius Kabi AB, Uppsala, Sweden) 15 min after sedation. All the dogs were preoxygenated before tracheal intubation. When palpebral and gag reflexes were absent, a tracheal tube was placed. Anesthesia was maintained with sevoflurane (SVO; Dechra Pharmaceuticals, Northwich, United Kingdom) (vaporizer 2.00–2.50%) in pure oxygen (2.00 L min^−1^) 25 min after sedation. A 0.9% sodium chloride solution was administered at a flow rate of 10.00 mL kg^−1^ per hr with an infusion pump (Braun, Melsungen, Germany). Dogs were placed in a dorsal recumbency, and after trichotomy, abdominal skin was prepared aseptically. The surgical procedures were started 40 min after sedation. Surgery time was defined as the time from skin incision to skin closure. Anesthesia time was defined as the time from sedation until the termination of inhalation of SVO. Ketoprofen at a dose of 2.00 mg kg^−1^ (KET; Merial, Toulouse, France) was administered intravenously to all dogs after the surgery.

### 2.2. Analysis of Blood Samples

**Figure 1:**
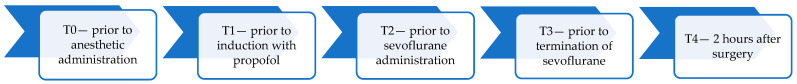
Visual timeline of different blood sample collection timings.

Venous blood samples (3.00 mL) were collected into heparin tubes via venipuncture from the jugular vein. Blood samples were taken at five time points: T0—before sedation with the administration of drugs (MEDBUT/MEDBUP); T1—before anesthesia induction with propofol (t = 15 min); T2—before attaching the animal to the anesthesia machine and maintenance with sevoflurane (t = 25 min); T3—before disconnecting the tracheal tube from the anesthesia machine and terminating sevoflurane administration (t = 1 h 50 min); T4—at 2 h after the surgery (t = 3 h 50 min) (Figure 1). Plasma samples were separated by centrifugation at 1500 rpm for 15 min and were stored at −80.00 °C until analysis. Total oxidant status (TOS) and total antioxidant status (TAS) were determined by using a Lambda 25 UV/Vis spectrophotometer (PerkenElmer, Waltham, MA, USA) and Rel Assay Diagnostics kits (Mega Tip, Gaziantep, Turkey) following the manufacturers’ instructions as proposed by Erel, 2005 [10]. The oxidative stress index (OSI) was calculated as follows: OSI (arbitrary unit) = TOS (μmol HO_2_ Eq L^−1^)/TAS × 10 (mmol Trolox Eq L^−1^) [11].

### 2.3. Monitoring

Heart rate (HR; beats min^−1^); oxygen saturation (SpO_2_; %); breathing rate, (fR; breaths min^−1^); end-tidal carbon dioxide (EtCO_2_; mmHg); systolic arterial pressure (SAP; mmHg); and diastolic arterial pressure (DAP; mmHg) were monitored by using a veterinary patient monitoring system (iM8 VET, Edan, Langen, Germany). Body temperature (BT; °C) was measured using an electric thermometer. SAP and DAP were measured using a non-invasive oscillometric method. SpO_2_ was measured by a pulse oximeter (OXY-100 VET, Gima, Fara d’Adda, Italy), and EtCO_2_ was measured using a capnograph, installed into an anesthesia machine (WATO EX-35, Mindray, Shenzhen, China). An electronic circulating warm heating pad (MHP-E1220; Anpan, Shenzhen, China) was used to maintain BT between 38.20 and 39.30 °C. Anesthesia depth and animals’ vital parameters were continuously monitored every 5 min by an anesthesiologist who was blinded to the used treatment. HR, fR, SAP, DAP, SpO_2_, and BT were also measured 2 h after surgery. The first vital parameter evaluation at t = 5 min was used as a control to compare the latter vital parameter calculations.

### 2.4. Statistical Analysis

The data normality was evaluated by the Shapiro–Wilk test. The majority of the data were distributed normally; therefore, mean ± standard deviation (SD) is furtherly used to describe the values. Statistical analysis was performed with the two-tailed Student’s *t* test. A Confidence Interval (CI) of 95% was used in the statistical analysis. The data were considered statistically significant if *p*-value < 0.05. Calculations were performed by using SigmaPlot 10.0 software (Systat Software GmbH, Erkrath, Germany).

## 3. Results

Both the MEDBUT and MEDBUP groups were matched for age (15.0 ± 8.0 and 21.2 ± 8.8 months, respectively) and weight (25.8 ± 5.9 and 20.1 ± 3.1 kg, respectively) (*p* < 0.05). The total amount of propofol injected in the MEDBUT and MEDBUP groups was 1.8 ± 0.3 and 0.99 ± 0.5 mg kg^−1^, respectively. The ovariohysterectomy lasted for 1 h 10 (±10) min in total, and the anesthesia time (T0–T3) lasted 1 h 50 (±10) min for all examined dogs in this study. Initial physiological and vital parameters before the procedure did not statistically differ in the MEDBUT and MEDBUP animal groups (*p* < 0.05). All dogs were stable throughout the procedure and completed the study.

### 3.1. Oxidative Stress Evaluation

**Figure 2:**
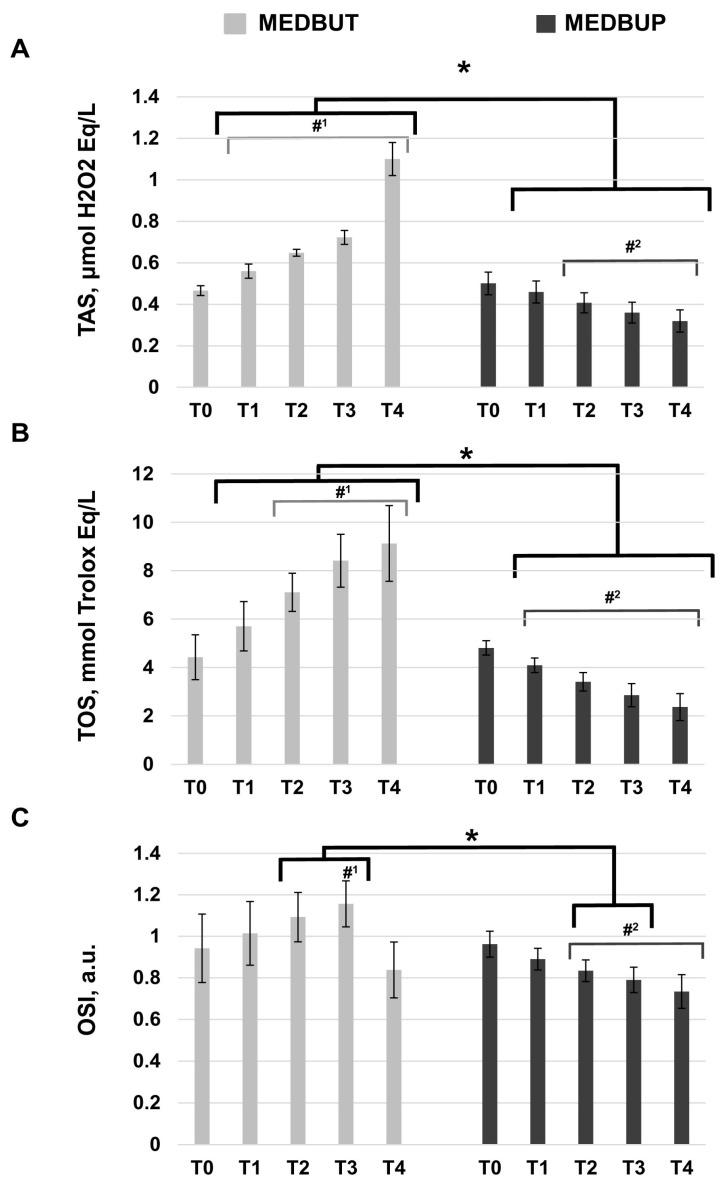
OS alterations in the dogs sedated with medetomidine–butorphanol (MEDBUT) and medetomidine–buprenorphine (MEDBUP) at different time points of the study: (**A**) Total antioxidant status (TAS), (**B**) total oxidant status (TOS), (**C**) oxidative stress index (OSI); propofol and sevoflurane was used in both examined groups for the induction and maintenance of anesthesia, respectively. Values are expressed as means ± standard deviation. (T0)—blood sample taken 1 min before sedation, control; (T1)—blood sample taken before the induction of anesthesia (15 min after sedation); (T2)—blood sample taken before connecting the dog to the anesthesia machine (25 min after sedation); (T3)—blood sample taken at the end of the study before SVO termination (1 h 50 min after sedation); (T4)—blood sample 2 h after surgery. #1 Significantly different from control (T0) within MEDBUT group (*p* < 0.05). #2 Significantly different from control (T0) within MEDBUP group (*p* < 0.05). * Significantly different between MEDBUT and MEDBUP groups at the same time points (*p* < 0.05).

The effect of MEDBUT and MEDBUP on plasma TAS, TOS, and OSI levels at different time points (T0–T4) is demonstrated in Figure 2. In the MEDBUT group, the TAS and TOS levels were increasing during T1–T4 (Figure 2A,B). The TAS value increased significantly from 0.47 ± 0.02 mmol Trolox Eq L^−1^ at T0 to 1.10 ± 0.08 mmol Trolox Eq L^−1^ at T4 (*p* < 0.05) and TOS from 4.42 ± 0.9 μmol H_2_O_2_ Eq L^−1^ at T0 to 9.13 ± 1.6 μmol H_2_O_2_ Eq L^−1^ at T4 (*p* < 0.05). Subsequent changes in the OSI are demonstrated in Figure 2C. The OSI level in the MEDBUT group increased significantly by 23% at T3 compared to the control (from 0.94 ± 0.1 to 1.16 ± 0.1 arbitrary units (a.u.)) (*p* < 0.05). At T4, the OSI value (0.84 ± 0.1 a.u.) significantly decreased in comparison to the control level (*p* < 0.05). In the MEDBUP group, an opposite tendency was observed for OS parameters. The TAS decreased from 0.50 ± 0.05 mmol Trolox Eq L^−1^ at T0 to 0.32 ± 0.05 mmol Trolox Eq L^−1^ at T4 (*p* < 0.05), and TOS from 4.81 ± 0.3 μmol H_2_O_2_ Eq L^−1^ at T0 to 2.37 ± 0.6 μmol H_2_O_2_ Eq L^−1^ at T4 (*p* < 0.05). The OSI level in the MEDBUP group significantly decreased by 27% at T4 compared to the control value (*p* < 0.05) (Figure 2C). The comparative analysis revealed significant differences between the MEDBUP and MEDBUT groups in TAS and TOS levels at the T1–T4 time frames; meanwhile, differences in OSI levels were observed at T2 and T3 with significantly higher values in the MEDBUT group.

### 3.2. Cardiorespiratory Parameter Evaluation

**Figure 3:**
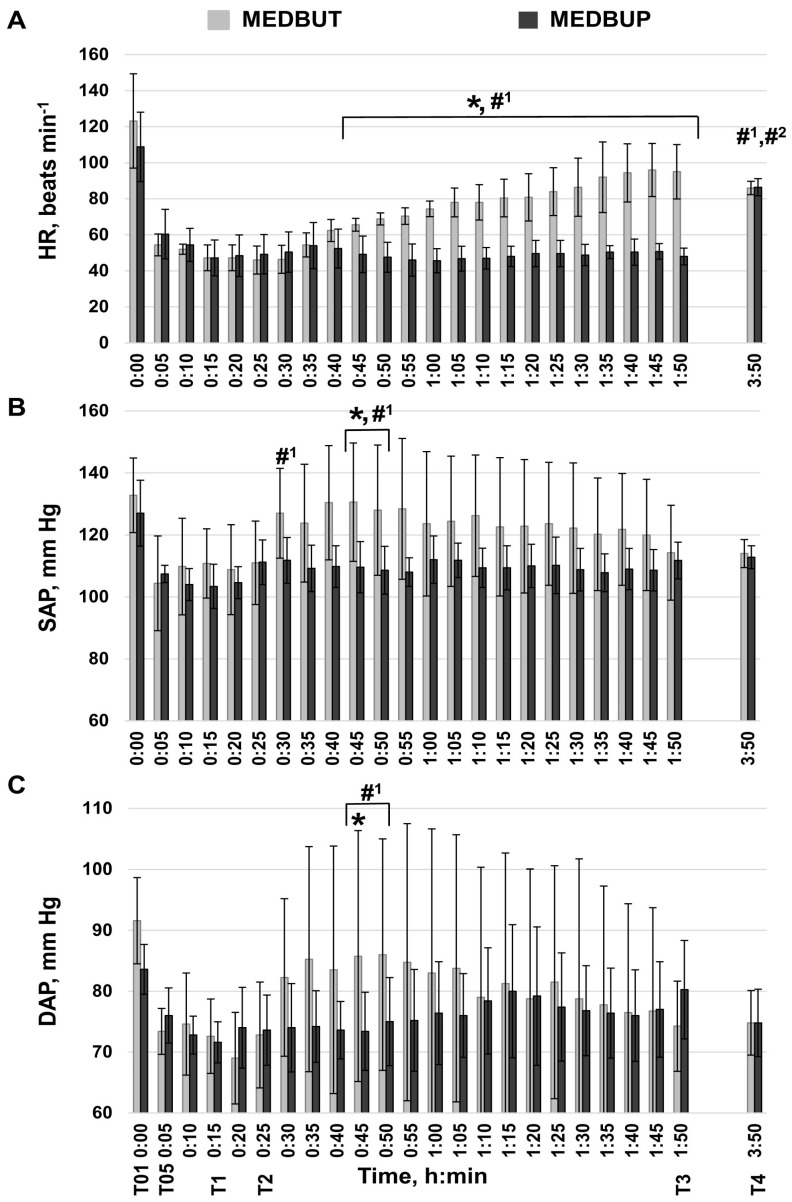
Cardiovascular parameter dynamic changes in the dogs anesthetized with medetomidine–butorphanol (MEDBUT) and medetomidine–buprenorphine (MEDBUP) at different time points in the study: (**A**) Heart rate (HR), (**B**) non-invasive systolic (SAP) and (**C**) diastolic (DAP) blood pressure; propofol and sevoflurane were used in both examined groups for the induction and maintenance of anesthesia, respectively. Values are expressed as means ± standard deviation. (T01)—time of starting sedation with MEDBUT or MEDBUP; (T05)—5 min after sedation, control; (T1)—time before the induction of anesthesia (15 min after sedation); (T2)—time before connecting the dog to the anesthesia machine (25 min after sedation); (T3) time before SVO termination, the end of the surgery (1 h 50 min after sedation); (T4)—2 h after surgery, at the end of the study. #1 Significantly different from control (T05) within the MEDBUT group (*p* < 0.05). #2 Significantly different from control (T05) within the MEDBUP group (*p* < 0.05). * Significantly different between MEDBUT and MEDBUP groups at the same time points (*p* < 0.05).

**Figure 4:**
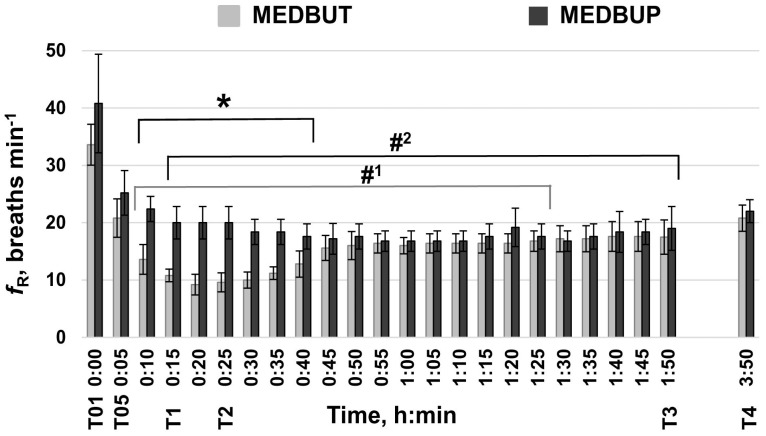
Respiratory rate (fR) dynamic changes in the dogs anesthetized with medetomidine–butorphanol (MEDBUT) and medetomidine–buprenorphine (MEDBUP) at different time points in the study; propofol and sevoflurane were used in both examined groups for the induction and maintenance of anesthesia, respectively. Values are expressed as means ± standard deviation. (T01)—time of starting sedation with MEDBUT or MEDBUP; (T05)—5 min after sedation, control; (T1)—time before the induction of anesthesia (15 min after sedation); (T2)—time before connecting the dog to the anesthesia machine (25 min after sedation); (T3)—time before SVO termination, the end of the surgery (1 h 50 min after sedation); (T4)—2 h after surgery, the end of the study. #1 Significantly different from control (T05) within the MEDBUT group (*p* < 0.05). #2 Significantly different from control (T05) within the MEDBUP group (*p* < 0.05). * Significantly different between MEDBUT and MEDBUP groups at the same time points (*p* < 0.05).

Cardiovascular (HR, SAP, DAP) and respiratory (fR, SpO_2_) parameters and BT were continuously monitored during the procedure (T01–T3) and 2 h post-operatory (T4). EtCO_2_ was registered at the T2–T3 time frames. BT and SpO_2_ remained at the baseline levels during all procedures and did not differ between the examined animal groups. EtCO_2_ did not differ at T2–T3 (Table 1). Higher variability in HR, fR, SAP, and DAP parameters was observed in dogs after the administration of MEDBUT compared to dogs sedated with MEDBUP. These parameters were stable until T4 in the MEDBUP group (Figure 3 and Figure 4), except for the HR value at the end of the study (Figure 3A). At t = 5 min, monitored HR was 60 ± 14 beats min^−1^, and similar rates (below 70 beats min^−1^) remained during T0–T3. At the end of the study (T4), animals reached physiologic HR.

In the MEDBUT group, HR decreased to 54 ± 6 beats min^−1^ at t = 5 min with no alterations until the 40th min. Afterwards, it started to increase and reached 95 ± 13 beats min^−1^ at T3 (*p* < 0.05). At the end of the study (T4), monitored HR was 86 ± 4 beats min^−1^. Relevant dynamics of SAP and DAP are presented in Figure 3B,C. SAP and DAP decreased after sedation with both MEDBUT and MEDBUP agents. SAP and DAP had returned to the initial values (T0) by the 30th min of the procedure in the MEDBUT group. In contrast, in the MEDBUP group, the SAP and DAP values reached at t = 5 min (107.4 ± 2.8 and 76 ± 4.5 mmHg, respectively) remained unchanged during the whole procedure. At the end of the study (T4), the SAP and DAP values were similar to the control values (t = 5 min) and did not differ between the MEDBUT and MEDBUP groups. The monitored SAP and DAP values were 114 ± 4.5 mmHg and 74.8 ± 5.3 mmHg in the MEDBUT group and 112.8 ± 3.7 mmHg and 74.8 ± 5.5 mmHg in the MEDBUP group, respectively.

Similarly to SAP and DAP parameters, a decrease in fR in both examined groups after the administration of sedative agents was documented (Figure 4). However, a steeper decline in fR early in the study after MEDBUT administration led to significant differences between the examined animal groups. In the 20th min, the fR in the MEDBUT group was almost two times lower than that in the MEDBUP group (9.2 ± 1.8 and 20 ± 2.8 breaths min^−1^, respectively) (*p* < 0.05). At T4, fR values did not differ when compared to control values (*p* < 0.05).

## 4. Discussion

Although the topic has been largely studied before, the results of different anesthetic agents’ effects on oxidative stress are confusing. The veterinary literature states that butorphanol has mostly antioxidant effects [28,29]. Kang et al. concluded that butorphanol slowed down radical attacks against B-phycoerythrin as an antioxidant [22]. The combination of fentanyl and butorphanol was reported to have antioxidant and cardioprotectant features in rats [30]. In our study, the OSI level in the MEDBUT group increased by 23% at T3 (1 h 50 min after sedation) in comparison to the control. At T4 (2 h after surgery), the oxidative stress index significantly decreased, becoming lower than the control value. Despite this, in our study, the OSI levels at T2 and T3 were significantly higher in the MEDBUT group.

Results regarding buprenorphine’s antioxidant properties are less unanimous. Kang et al.’s study reported that buprenorphine, as a powerful antioxidant, did not just slow down but completely stopped and prevented radical attacks against B-phycoerythrin in vitro [22]. Buprenorphine exposure in utero led to increased antioxidant activity and inhibited apoptosis in the hippocampi of rat pups [32]. However, when buprenorphine was used for opioid maintenance treatment, it caused severe oxidative stress and cognitive deficits in humans [20]. In mouse arthritis models, buprenorphine administration significantly increased inflammatory mediator production and was associated with important oxidative action [6]. Buprenorphine also caused significant oxidative damage to rats’ livers by increasing lipid peroxidation, reducing the amount of antioxidant enzymes and causing elevation in hepatic biomarkers [31]. In the current report, MEDBUP-group dogs’ TAS, TOS, and OSI levels decreased throughout the study, with control values being the highest. The oxidative stress index level significantly decreased by a total of 27% from T0 to T4.

Our results mildly differ from already-reported findings. This study suggests that even if the literature is controversial, buprenorphine might have superior antioxidant capacities and could potentially prevent oxidative damage during anesthesia for routine surgeries. Butorphanol, on the other hand, demonstrated mild oxidative action, contrary to the previous reports available. It remains possible that buprenorphine displays better antioxidant properties than butorphanol because the latter has a shorter duration of action and is indicated for painless or mild-to-moderate-pain-causing procedures [12]. Even if it is not likely, pain stress could have been higher in the MEDBUT group. Antioxidants like melatonin could be administered as prophylaxis to reduce or prevent oxidant effects during OHE [35].

Butorphanol and buprenorphine were also previously compared as anesthetic agents in multiple studies, in which the results differed widely [36,37,38,39,40,41,42]. Opioid drug sedation is more effective if combined with alpha 2-adrenergic agonists [36,37]. For example, medetomidine–buprenorphine (MEDBUP) combination was reported to be an effective and convenient sedation option in dogs [38]. Warne et al. also reported that buprenorphine provides adequate analgesia, while all patients in the BUT group needed rescue analgesia during a routine OHE in cats [39]. But, in later studies, it was concluded that dexmedetomidine–butorphanol (DEXMEDBUT) provides superior sedation in cats compared to dexmedetomidine–buprenorphine (DEXMEDBUP) [40]. The DEXMEDBUT protocol was also superior to DEXMEDBUP combination while performing hip radiography in dogs [33]. The MEDBUT anesthetic combination did not even significantly differ from methadone–medetomidine [41]. Gültiken et al., on the other hand, concluded that butorphanol might not provide sufficient analgesia even in routine surgical procedures [29]. Anesthesia and its efficiency are usually evaluated by measuring cardiorespiratory parameters. The administration of both medetomidine and butorphanol was correlated with increases in SAP, MAP, DAP, and BT and decreases in HR and RR, displaying cardiopulmonary effects [37]. Both MEDBUT and MEDBUP displayed significant increases in blood pressure (BP) and decreases in cardiorespiratory parameters (HR, fR) and BT [38,42]. Usually, negative side effects like bradycardia and hypertension or hypotension in anesthetized animals are a consequence of alpha 2-adrenergic agonist action [28,37,41]. These effects can also be seen in our results, as all HR, SAP, DAP, and fR values dropped after sedation administration. Butorphanol was more likely to increase BP and heart rate during the study. Overall, MEDBUP values tended to be more stable and changed less than those in the MEDBUT group.

It has been reported that propofol decreases arterial BP and does not change heart rate or oxygen delivery but can cause apnea, hypoventilation, or hypoxemia [43,44]. In our study, propofol was administered at t = 15 min, but no changes were noted in the observed parameters.

Sevoflurane was reported to increase heart rate and cause systemic vasodilation and hypotension while preserving hepatic blood flow [45,46]. Although research states that the percentage of oxygen delivered during anesthesia does not influence oxidative stress, we delivered sevoflurane in pure oxygen, ensuring equal conditions for all patients [47]. Sevoflurane began to be administered at t = 25 min, but no correlations were made with its use.

Reportedly, castration in male dogs and horses can cause pain stress and oxidative stress and decrease overall antioxidant power [48,49,50]. Pain stress can be expressed as an elevation of heart and respiratory rate in dogs after castration [48]. Although not very likely, it remains possible that the elevation of heart rate and hypertension observed in the MEDBUT group could be a consequence of pain stress or inadequate analgesia.

There are several limitations in the present study. First, a limited number of patients were enrolled into the study. Additionally, pain score was not evaluated in this study, as our primary goal was not the comparison of intraoperative analgesia using different anesthetic protocols. Lastly, various interactions and multifactorial environmental influences on oxidant and antioxidant powers must be considered. The lack of test sensitivity when evaluating oxidative stress can mean that the influences of butorphanol and buprenorphine on OSI were not necessarily reflected in this study.

## 5. Conclusions

In conclusion, buprenorphine might have superior antioxidant capacities and could potentially prevent oxidative damage during anesthesia for routine OHE. Butorphanol demonstrated mild oxidative action in this study. All dogs’ cardiorespiratory parameters were stable throughout the procedure and all animals recovered uneventfully. The HR, SAP, DAP, and fR values dropped after sedative administration in both groups but were altered at significantly greater levels in animals sedated by MEDBUT. This might indicate that buprenorphine is an overall superior anesthetic agent compared to butorphanol for dogs undergoing OHE.

## Figures and Tables

**Table 1:** Effects of sedation with medetomidine–butorphanol (MEDBUT) and medetomidine–buprenorphine (MEDBUP) on saturation of peripheral oxygen (SpO_2_), end-tidal CO_2_ (EtCO_2_), and body temperature (BT) at different time points in the study; propofol and sevoflurane were used in both examined groups for the induction and maintenance of anesthesia, respectively.

Time (hr:min)	SpO_2_ (%)		EtCO_2_ (mmHg)		BT (°C)	
	MEDBUT	MEDBUP	MEDBUT	MEDBUP	MEDBUT	MEDBUP
00:00	100 ± 0	100 ± 0	-	-	38.9 ± 0.5	39.0 ± 0.2
00:05	100 ± 0 *	100 ± 0 *	-	-	39.1 ± 0.4 *	39.2 ± 0.2 *
00:10	100 ± 0	100 ± 0	-	-	39.1 ± 0.4	39.2 ± 0.1
00:15	100 ± 0	100 ± 0	-	-	39.2 ± 0.3	39.2 ± 0.1
00:20	100 ± 0	100 ± 0	-	-	39.1 ± 0.2	39.1 ± 0.1
00:25	100 ± 0	100 ± 0	46.8 ± 3.3 *	40.8 ± 10.2 *	39.1 ± 0.3	39.1 ± 0.2
00:30	100 ± 0	100 ± 0	46.6 ± 7.2	42.0 ± 5.8	39.0 ± 0.2	39.1 ± 0.1
00:35	100 ± 0	100 ± 0	45.4 ± 6.0	42.0 ± 7.1	39.0 ± 0.3	39.1 ± 0.2
00:40	100 ± 0	100 ± 0	45.6 ± 4.5	40.8 ± 6.1	38.9 ± 0.2	39.0 ± 0.2
00:45	100 ± 0	100 ± 0	43.4 ± 5.1	41.6 ± 6.4	38.9 ± 0.3	38.9 ± 0.1
00:50	100 ± 0	100 ± 0	44.4 ± 4.4	40.4 ± 5.7	38.8 ± 0.3	38.9 ± 0.2
00:55	100 ± 0	100 ± 0	45.2 ± 4.3	41.4 ± 6.2	38.8 ± 0.3	38.9 ± 0.2
01:00	100 ± 0	100 ± 0	45.2 ± 4.5	39.4 ± 6.1	38.7 ± 0.3	38.8 ± 0.3
01:05	100 ± 0	100 ± 0	42.4 ± 5.5	40.6 ± 5.4	38.7 ± 0.3	38.8 ± 0.3
01:10	100 ± 0	100 ± 0	44.8 ± 5.5	41.6 ± 7.3	38.7 ± 0.3	38.7 ± 0.3
01:15	100 ± 0	100 ± 0	43.8 ± 5.9	41.0 ± 5.7	38.6 ± 0.3	38.6 ± 0.3
01:20	100 ± 0	100 ± 0	45.4 ± 5.9	39.2 ± 6.3	38.7 ± 0.3	38.6 ± 0.4
01:25	100 ± 0	100 ± 0	45.6 ± 5.9	38.2 ± 5.6	38.6 ± 0.3	38.5 ± 0.4
01:30	100 ± 0	100 ± 0	45.8 ± 5.9	39.2 ± 6.1	38.6 ± 0.3	38.5 ± 0.4
01:35	100 ± 0	100 ± 0	44.8 ± 8.0	38.8 ± 6.8	38.6 ± 0.3	38.4 ± 0.4
01:40	100 ± 0	100 ± 0	43.8 ± 7.9	39.4 ± 6.0	38.6 ± 0.3	38.4 ± 0.4
01:45	100 ± 0	100 ± 0	45.4 ± 5.7	38.8 ± 6.3	38.6 ± 0.3	38.4 ± 0.4
01:50	100 ± 0	100 ± 0	44.75 ± 6.7	39.0 ± 7.0	38.4 ± 0.1	38.4 ± 0.5
03:50	100 ± 0	100 ± 0	-	-	38.4 ± 0.1	38.3 ± 0.1

* There were no significant differences as compared to control (t = 5 min for SpO_2_ and BT, t = 25 min for EtCO_2_). * (*p* > 0.05).

## Data Availability

The data presented in this study are available on request from the corresponding author upon reasonable request.
